# Nano-Hydroxyapatite Composite Scaffolds Loaded with Bioactive Factors and Drugs for Bone Tissue Engineering

**DOI:** 10.3390/ijms24021291

**Published:** 2023-01-09

**Authors:** Xiaojing Mo, Dianjian Zhang, Keda Liu, Xiaoxi Zhao, Xiaoming Li, Wei Wang

**Affiliations:** 1 Liaoning Provincial Key Laboratory of Oral Diseases, School and Hospital of Stomatology, China Medical University, Shenyang 110001, China; 2 Key Laboratory for Biomechanics and Mechanobiology of Ministry of Education, Beijing Advanced Innovation Center for Biomedical Engineering, School of Biological Science and Medical Engineering, Beihang University, Beijing 100083, China

**Keywords:** nano-hydroxyapatite, composite scaffold, bioactive factor, drug delivery, bone regeneration

## Abstract

Nano-hydroxyapatite (n-HAp) is similar to human bone mineral in structure and biochemistry and is, therefore, widely used as bone biomaterial and a drug carrier. Further, n-HAp composite scaffolds have a great potential role in bone regeneration. Loading bioactive factors and drugs onto n-HAp composites has emerged as a promising strategy for bone defect repair in bone tissue engineering. With local delivery of bioactive agents and drugs, biological materials may be provided with the biological activity they lack to improve bone regeneration. This review summarizes classification of n-HAp composites, application of n-HAp composite scaffolds loaded with bioactive factors and drugs in bone tissue engineering and the drug loading methods of n-HAp composite scaffolds, and the research direction of n-HAp composite scaffolds in the future is prospected.

## 1. Introduction

Repairing of large segmental bone defects caused by severe trauma, osteoporosis, tumor resection and bone infection is one of the most difficult challenges for orthopedic surgeons [1,2]. The major solutions for these large bone defects are autologous bone grafting, allogeneic bone grafting and synthetic bone substitutes [3]. At present, autologous bone grafting still has many problems, such as secondary trauma to patients and donor site morbidity [4,5]. In addition, the allogeneic bone possesses a number of limitations, such as reduced biological activity, immune rejection and pathogen transmission [6,7]. Therefore, researchers have been working on making ideal bone scaffolds similar to natural bone, such as high mechanical strength and suitable pore size and porosity, which can promote proliferation, migration and differentiation of osteoblasts while achieving the purpose of support [8]. Hydroxyapatite (HAp), Ca_10_(PO_4_)_6_(OH)_2_, with structural and biochemical properties similar to the mineral composition of human bone tissue, was regarded as a promising implant material in the 1970s [9,10]. It is one of the anticipated biomaterials for bone tissue engineering and regeneration with excellent biocompatibility, high osteoconductivity, osteoinductivity and biological activity [11,12,13]. In addition, nano-hydroxyapatite (n-HAp) with a particle size of 1–100 nm possesses favorable advantages of higher surface area to promote osteoblast function, close contact with surrounding tissues, high biological activity and overcomes the issue of low biodegradability [14,15]. Considering that high brittleness and powdery characteristics of n-HAp make it difficult to use alone in bone reconstruction sites, many researchers have combined it with various materials, such as natural and synthetic organic materials, to form composite biomaterials [16,17] in order to remedy the problems of insufficient fracture toughness, fatigue failure and low mechanical strength of pure n-HAp biomaterials [18].

**Figure 1:**
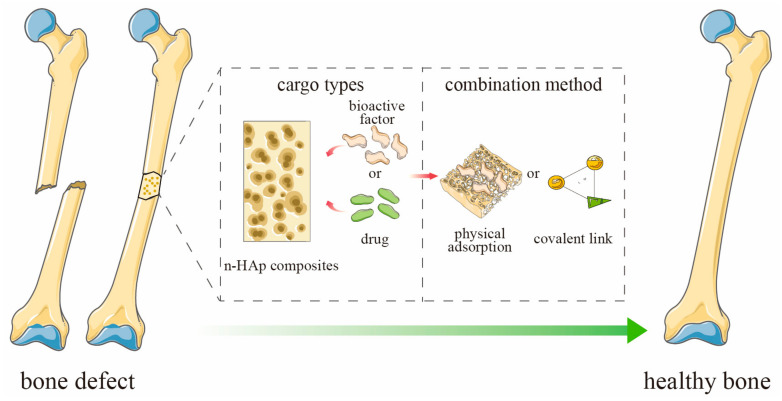
Schematic representation of n-HAp composite scaffold loaded with bioactive factors or drugs for bone defect.

In contemporary medicine, bone engineering is committed to creating a bionic system through coordinated combination of cells, scaffolds and bioactive factors to repair damaged bone tissue [19]. Controlled delivery of bioactive factors in a biological scaffold carrier can promote and accelerate functional bone formation [20]. Thus, synthetic scaffold materials loaded with bioactive factors that are used in tissue engineering technology to aid regeneration of new tissues or organs in vivo has become a research hotspot in reconstruction of bone defects [21]. Composites based on n-HAp can meet the conditions of existing bone tissue engineering artificial bone. One promising approach is modification of scaffold materials with bioactive factors with an osteogenic effect [22,23]. Further, n-HAp composite scaffolds provide support for cell growth, migration and differentiation, and these bioactive factors are an important component of simulating the bone microenvironment in a bone tissue engineering system, which can improve the bioactivity of n-HAp surface. They not only attract and aggregate surrounding stem cells to the bone defect area but also promote osteogenic differentiation of the original stem cells [24,25]. As a transportation system for bioactive factors, a scaffold can ensure their stable and sustained release, preserve their biological activity and simulate the natural bone microenvironment [19]. In recent years, there has been strong interest in bone-targeted therapy based on scaffold materials due to the increasing number of patients requiring pharmacological treatment for bone tissue disorders [26,27]. Indeed, n-HAp composite scaffolds can become a promising option as a targeted delivery system based on bone tissue engineering scaffold and possess superiority to n-HAp, involving great osteoconductivity and supporting cellular responses for bone reconstruction, as well as upgrading the poor mechanical property of n-HAp [23,28]. By adding various bioactive factors or different drugs to bone scaffolds, the regenerative capacity of bone tissue can be enhanced and bone tissue diseases, such as osteomyelitis and bone tumors, can be treated locally (Figure 1) [29,30,31]. Further, n-HAp-based composite scaffolds are widely used as carriers for various reagents and have become one of the hot spots in the field of medical materials.

This review summarizes the recent advances in n-HAp composites for bone defect repair from the following aspects. First, the n-HAp composites are classified and elaborated. Second, application of n-HAp composites as carriers for loading bioactive factors and different drugs in bone tissue engineering is introduced. Third, the different methods of loading bioactive factors and drugs on n-HAp composites are summarized. Finally, the future research directions of n-HAp composite bone scaffolds are prospected.

## 2. Structure and Properties of n-HAp

**Figure 2:**
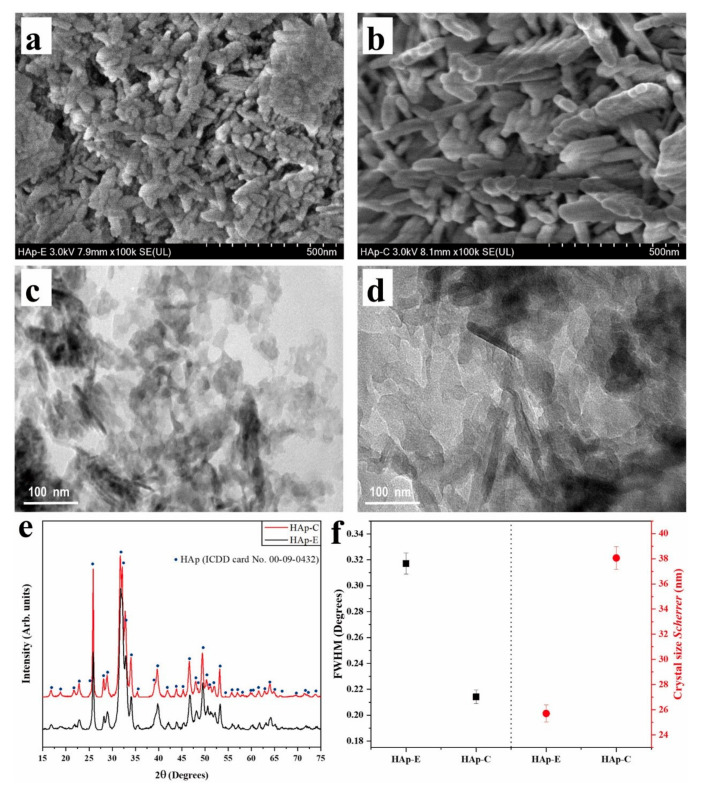
HR-SEM images of (**a**) HAp-E and (**b**) HAp-C at 100,000×. HR-TEM images of (**c**) HAp-E and (**d**) HAp-C at 100,000×. X-ray diffraction patterns of (**e**) HAp-E and HAp-C. (**f**) FWHM and crystal size by Scherrer’s equation [42]. Reproduced with permission from copyright © 2022, ELSEVIER.

HAp is an important bone regeneration material in clinical applications and is usually used as a filler to promote bone regeneration, so it is necessary to simulate the physicochemical properties of HAp in human bone, such as the size and morphology of crystals [32]. HAp in human bone consists of highly ordered crystals at the nanoscale [33]. Further, n-HAp can be synthesized from chemical precursors or extracted from natural resources or biological waste, such as eggshells, animal bones and some plants [34]. Acquisition of synthetic and biogenic HAp usually requires calcination at high temperatures to remove reaction by-products. During the calcination process, the morphology and structure of HAp will change. As the calcination temperature increases, HAp derived from pig bone will experience three mass losses and one mass gain, which are related to dehydration, organic phase degradation, carbonate loss and oxide formation, respectively [35]. Another study showed that calcining HAp at a higher temperature also observed the appearance of β-tricalcium phosphate (β-TCP), which may be related to loss of hydroxyl groups [36]. Studies have pointed out that, when the calcination temperature is higher than 700 °C, HAp will change from nanocrystals to microcrystals under the influence of temperature, which will affect its clinical application [37,38]. The crystal structure of HAp can affect its electrical properties [39], and electrical properties are related to cell proliferation and fracture healing, and a higher dielectric constant contributes to cell proliferation [36,40]. Ramirez-Gutierrez et al. used in situ impedance spectroscopy (IS) to study the electrical properties of HAp derived from porcine bone and correlate them with the structural and thermal transitions observed by differential scanning calorimetry (DSC) and high-temperature X-ray diffraction (HT-XRD). The results of DSC, HT-XRD and IS showed that, at 700 °C, the HAp crystals transformed from nanoscale to microscale, and magnesium (Mg) expanded outward to the surface to form magnesium oxide (MgO). These processes improve the quality of the crystal and the activation energy of the second cycle, which confirm that the crystal structure is closely related to the electrical properties [35]. Gittings et al. investigated the electrical properties of synthetic HAp sintered at different temperatures and atmospheres. The results showed that the permittivity and alternating current conductivity of HAp were related to the thermal history at 700 °C. At higher temperatures (700 °C–1000 °C), the conductivity of HAp is no longer affected by thermal history but closely related to conduction of hydroxyl ions (OH^−^) [41]. In order to avoid conversion of synthesized HAp crystals to the micron size, Gomez-Vazquez et al. used eggshells as raw materials to obtain biomimetic nano-hydroxyapatite (HAp-E) by wet chemical method at a sintering temperature of 250 °C. As shown in Figure 2, high-resolution scanning electron microscopy (HR-SEM), high-resolution transmission electron microscopy (HR-TEM) and X-ray diffraction (XRD) images confirmed that the crystal size of the synthesized HAp was nanoscale. Compared with the commercial sample (HAp-C) with a crystal size of 25–255 nm, the size of HAp-E is smaller at 10–90 nm. The morphological differences between HAp-E and HAp-C may be related to the roasting temperature and a small amount of ions from eggshell [42]. Studies have shown that mineral elements can affect the morphological structure of HAp during sintering. The research results of Ramirez-Gutierrez et al. showed that, as the sintering temperature increased, the Mg in the pig bone diffused outward to form MgO and the HAp crystals became faceted, improving the crystal quality [43]. Londoño-Restrepo et al. also came to a similar conclusion that, when Mg diffuses out of the lattice of HAp, it can improve the order and crystal quality within the lattice [44]. Pal et al. used *Mercenaria clam shells* as raw materials to prepare strontium-doped hydroxyapatite by hydrothermal method to understand the effect of strontium (Sr) incorporation on HAp sintering behavior, phase stability and mechanical properties. Addition of Sr activates the sintering process, and the sintered samples showed smaller grain size, increased density and improved mechanical properties [45].

## 3. n-HAp Composite Scaffolds for Bone Repair

Novel materials made by physical or chemical methods from the composite of two or more materials with different properties are known as composites. Simple n-HAp biomaterials have problems, such as insufficient fracture toughness, fatigue failure and low mechanical strength, which limit their clinical applications [18]. Several studies have combined n-HAp with other biological materials to improve the performance of n-HAp. Biomaterials can be divided into two categories according to their chemical composition: natural polymer materials and synthetic polymer materials.

### 3.1. n-HAp/Natural Polymers Composite Scaffolds

Chitosan (CS) is a cationic natural polymer made from natural polysaccharide chitin by removing some of its acetyl groups [46,47] and is considered a potential polysaccharide for development of bone tissue engineering scaffolds due to its obvious advantages, such as good biocompatibility, biodegradability, non-toxicity, antibacterial ability, biofunctionality and low immunogenicity [48]. In modern medicine, although chitosan has been widely used in bone tissue engineering, its weaknesses of poor mechanical character and instability have limited its applications in the load-bearing area. Consequently, chitosan requires addition of reinforcements to synthesis composites, such as n-HAp, which has been demonstrated to improve its mechanical strength [49,50]. In recent years, research efforts have focused on n-HAp/CS composites, a composite biological scaffold that combines enhanced mechanical properties and the desired properties of bio-functionality [51]. Previous studies have demonstrated that the high percentage content of n-HAp enhances the mechanical properties of these composite scaffolds; moreover, scaffolds of n-HAp/CS concentrations of 75/25 *w*/*w* have better physicomechanical properties, but an increase in hydroxyapatite concentration over 80% will lead to fragility of the scaffold in vitro [52]. A research group developed an n-HAp/CS scaffold with high n-HAp content (75% *w*/*w*) by freeze-drying and initially studied the mechanical properties of the scaffold [53]. The scaffold was then implanted into a rat skull defect model, and the amount of new bone tissue in the rat skull was evaluated by histological and histomorphometric analysis and cone beam computed tomography (CBCT) imaging [54]. The surface area of the residual biomaterial was used to evaluate the biodegradability of the n-HAp/CS scaffold [55]. These results indicate that, compared with pure chitosan scaffold, n-HAp/CS scaffold has better mechanical properties and biocompatibility and can provide enough space for new bone formation.

Silk fibroin (SF) is a natural polymer fibrin extracted from silk that has the characteristics of abundant hydrophilicity, controllable degradability, good biocompatibility, low immunogenicity and abundant sources [56,57]. However, the shortcomings of SF as a single material need to be modulated, such as weak osteoinductive and mechanical properties, making it insufficient to repair large bone defects. Thus, addition of n-HAp in SF was considered to synthesize composite scaffolds for bone regeneration, which can result in osteoconductivity, osteoinduction and enhance mechanical properties [58,59]. SF is demonstrated to play an important role in regulating the nucleation, crystal growth and mineralization of HAp. Shao et al. used microwave-assisted technology and different biomineralization methods to deposit n-HAp onto a porous SF scaffold to make a three-dimensional HAp/SF composite scaffold. This in vitro study proves that the compressive strength of the scaffold increases with an increase in HAp concentration, and it has compositions and a multi-layered structure similar to natural bone, which can provide a good cell survival microenvironment for bone repair [60]. Another in vivo study demonstrated that implantation of n-HAp/SF composite scaffolds can significantly enhance bone regeneration, which can lead to more fresh bone in alveolar bone and less reduction in alveolar bone height after tooth extraction [61]. As the inorganic and organic phases of many composites are simply mixed, there is little or no interaction between them. Therefore, Mobika et al. [62] used in situ co-precipitation method and Wei et al. [46] used Ca-P alternate soaking method and both of them prepared composite scaffolds with n-HAp particles uniformly distributed in SF, which improves cell adhesion and greatly promotes biomineralization. Researchers have utilized different means to prepare various n-HAp/SF composite scaffolds that possess desirable biocompatibility and bioactivity and have higher application value in the field of bone repair.

### 3.2. n-HAp/Synthetic Polymers Composite Scaffolds

Polylactic acid (PLA), also known as polylactide, is a biodegradable polymer with good biocompatibility, low immunogenicity, excellent mechanical properties and thermoplasticity, which has been approved by the US Food and Drug Administration for biomedicine [63,64]. However, the hydrophobic surface of PLA, lack of osteoinductivity, slow degradation rate and acidic products formed by hydrolysis that can trigger inflammation at the site of scaffold implantation have limited its application [65,66]. The combination of n-HAp and PLA can significantly increase the osteoinductivity and surface activity of the scaffold while maintaining the acid–base balance of the implant site [67]. Compared with some traditional preparation methods of porous scaffolds, such as solvent casting and freeze-drying, 3D printing technology allows for accurate structural control, which is more conducive to controlling the internal porosity, pore size and interconnected structure of materials [68]. Wang et al. successfully used fused deposition molding (FDM) technology to prepare filament and mud-like PLA/n-HAp composite scaffolds. The n-HAp is evenly distributed in the PLA. It can improve the hydrophilicity of the polymer and promote infiltration of water into the polylactic acid matrix, thus enhancing the degradation rate of the scaffold [69]. A group prepared porous PLA/n-HAp scaffold with pore diameter of 292 ± 1.8 μm by 3D printing technology. Its compressive strength reaches 23.36 ± 0.48 MPa. In vivo experiments demonstrated the ability of the scaffold to promote osteogenesis and osteoconductivity, while in vitro antibacterial experiments demonstrated its ability to load and release antibacterial drugs [67]. Hartatiek et al. prepared n-HAp/PLA composite scaffolds by adding different mass fractions of PLA into n-HAp matrix. The increase in PLA concentration reduced the porosity of the composite scaffolds. The best porosity of the composite scaffolds was achieved when the composition of n-HAp and PLA was 90:10 wt% [70].

Polycaprolactone (PCL) is widely used as a biodegradable material for medical purposes because of its good biodegradability, biocompatibility and non-toxicity [71]. However, its low biological activity, hydrophobicity and poor mechanical properties limit its application in bone tissue engineering [72,73]. In order to improve the mechanical properties of PCL, Juan et al. added n-HAp to the PCL matrix to form a three-dimensional porous composite scaffold with an elastic modulus of 0.401–1.005 GPa, within the range of elastic modulus of human cancellous bone (0.1– 4.5 GPa) [74,75]. The degradation rate of the bone tissue engineering scaffold should match the bone formation rate [76]. If the degradation rate is too fast or too slow, the implant will fail. A study characterized the performance of PCL/n-HAp complexes made from PCL of different molecular weights. The addition of n-HAp increases the viscosity and degradation rate of the composites [77]. Textile-based bone tissue engineering scaffolds made of composite fibers can mimic the extracellular matrix and provide sufficient pores for cell growth [78]. The fiber diameter of the PCL/n-HAp scaffold made by electrospinning technology is 211 ± 66 nm. Compared with pure PCL, the expression of osteopontin on the PCL/n-HAp scaffold increased by five times [79]. Furthermore, rotary jet spin (RJS) is a technology that uses centrifugal force to obtain micro/nano fiber materials, and its productivity is higher than that of electrospinning [80]. A research group used RJS to prepare PCL/n-HAp fiber scaffolds for orthopedic applications. With an increase in the content of n-HAp, the fiber diameter decreased from 1847 ± 1039 nm to 845 ± 248 nm, and the scaffold was shown to be non-cytotoxic but antimicrobial using a rat tibial defect model [81]. The PCL/n-HAp (20%) groups prepared by RJS were implanted in a severe defect area of the tibia of rats for 60 days, the initial formation of the Havers system can be observed and the biological performance of the new bone can be improved [82]. Table 1 summarizes the n-HAp composite scaffolds for bone regeneration.

## 4. Bioactive Factors-Loaded n-HAp Composite Scaffolds

The process of bone repair is complex, and bioactive factors play an important role in regulating osteoblast activity and bone regeneration [83]. Combining scaffolds with osteogenic bioactive molecules has become a mainstream strategy for bone tissue engineering [84]. In this section, we review recent research on use of n-HAp-based composites as carriers for the delivery of bioactive factors. These studies mainly include two parts: loading growth factors and synthetic peptides. Growth factors include bone morphogenetic protein (BMP), vascular endothelial growth factor (VEGF) and basic fibroblast growth factor (bFGF). The combination of n-HAp-based scaffolds with these bioactive factors can improve physiological activities, such as osteoinduction and angiogenesis, but controlled release, stability and bioactivity of bioactive factors are fundamental to successful growth factor therapy. Bioactive factors can be bound to scaffolds through physical adsorption or chemical coupling, resulting in sustained release [85].

### 4.1. Growth Factors

#### 4.1.1. BMPs

BMPs, also known as bone-forming proteins, are a group of highly conserved functional proteins with similar structures belonging to the transforming growth factor-β (TGF-β) family, which can induce formation of bone and cartilage in vivo [86]. Among them, well-known BMP-2 and BMP-7 have been approved by the FDA for clinical applications. MSc et al. composited polylactic acid-polyethylene glycol (PLA-PEG) with n-HAp to make a scaffold to support BMP-2. BMP-2 can be continuously released for 3 weeks in the composites and can well promote bone regeneration in spinal fusion models [17]. Growth factor release systems need to avoid the initial burst as it can lead to undesirable side effects, such as ectopic bone formation and immune responses [87]. Inspired by mussels, the catechol group in polydopamine forms a covalent binding with BMP-2, which enhances the connectivity between them and can reduce the initial burst release of BMP-2. Li et al. used polydopamine to immobilize BMP-2 on the polymer scaffold (PHB-PDA-BMP-2) [88]. BMP-2 in the PHB-PDA-BMP-2 scaffold sustained release for up to 30 days without an initial burst. Another study used a click-chemistry cross-linking mechanism to prepare dopamine-assisted silk fibroin hydrogels incorporating n-HAp-graphene oxide hybrid films and immobilize BMP-2 in the hydrogel [89]. The hydrogel scaffolds effectively transported BMP-2 to the bone defect site. Heparin can enhance the drug loading of BMP-2 the matrix by electrostatically binding with BMP-2 in a reversible manner. Yan et al. prepared Sr/n-HAp/SF scaffolds by freeze-drying and coupled heparin to load BMP-2. This BMP-2-loaded scaffold upregulated the activities of osteocalcin (OCN), osteopontin and alkaline phosphatase (ALP) and promoted new bone regeneration in vivo [90].

BMP-2 has the problems of short half-life, easy enzymatic hydrolysis in vivo and high synthesis cost [91]. Several studies have begun to explore other ways to promote bone healing. BMP-2-derived peptides are a good choice. BMP-2 contains a “wrist” epitope and a “knuckle” epitope that bind to BMP type I and type II receptors, respectively. Ye et al. synthesized a BMP-2 “knuckle” epitope-based polypeptide and immobilized it on an n-HAp/polylactic acid/gelatin scaffold. The BMP-2-derived peptides were continuously released in the scaffolds for 21 days and enhanced the osteogenic properties of the scaffolds [92]. BMP-2-mimicking peptide P28 is a polypeptide consisting of NH2- S[PO4]DDDDDDDKIPKASSVPTELSAISTLYL-COOH, which has been shown to promote the healing of rat calvarial defects [93]. Sun et al. loaded P28 on a bone scaffold composed of n-HAp/collagen/poly (L-lactide) (nHAC/PLA), and the results showed that P28/nHAC/PLA promoted cell migration, proliferation, differentiation and new bone formation at the femoral condyle defect site in rabbits [94]. Higher doses of BMP-2-derived peptides are required to produce similar osteogenic effects to BMP-2, which may be related to the presence of fewer BMP-2 receptor binding sites for the derived peptides. Future work requires more exploration of the BMP-2 receptor binding domain.

Aside from BMP-2, BMP-7, BMP-6 and BMP-9 were also loaded into scaffolds containing n-HAp and successfully improved the osteogenic properties of the scaffolds in vitro and in vivo. Li et al. combined BMP-7 transfected bone marrow mesenchymal stem cells with n-HAp/polyamide (n-HAp/PA) composite scaffolds to repair rabbit mandibular defects. The experimental results showed that there is more bone formation and early mineralization in the implanted area [95]. BMP-6 is a less studied member of the BMP family, and previous results have shown that slowly released BMP-6 promotes periodontal tissue regeneration and mineralization and healing of cranial defects in rats [96]. Another research group prepared gelatin microspheres loaded with BMP-6 and then combined them with n-HAp/gelatin scaffolds. The results show that BMP-6/nHAG/GMS scaffold has good biocompatibility and osteoinductive activity in vivo [84]. Zhang et al. prepared an n-HAp/collagen I (ColI)/multi wall carbon nanotube (MWCNT) composite scaffold and successfully loaded BMP-9. In vitro results showed that the composite scaffold promoted differentiation of bone marrow mesenchymal stem cells into osteoblasts. At the same time, in vivo results showed that the scaffold can induce more bone formation [97].

#### 4.1.2. VEGF

Vascular endothelial growth factor (VEGF) belongs to the family of pluripotent cytokines. In addition to promoting the migration, proliferation and angiogenesis of vascular endothelial cells, it also plays a role in formation and mineralization of osteoblasts in bone defects. Therefore, it can be combined with the scaffolds to improve the osteogenic efficiency [98]. Chen et al. modified HAp/calcium sulfate (HACS) scaffold with VEGF coating. In vitro study of HACS/VEGF scaffold confirmed that it could promote proliferation of hBMSCs and HUVECs, while in vivo study confirmed that it could significantly improve the regeneration and remineralization of bone tissue after 8 weeks of implantation in rabbit femoral defect [98]. Because physical adsorption technology and microsphere encapsulation technology can lead to a burst release and early delayed release of VEGF, Liu et al. have used bionic coprecipitation method to combine VEGF with 3D printed PCL/HAp scaffolds, which has a controllable release of VEGF, enhanced osteogenesis of stem cells and preeminent vascularized bone regeneration [99]. Furthermore, in order to enhance the controllability of VEGF, another study complexed VEGF and heparin onto the scaffold. This heparinised gelatine/hydroxyapatite/tricalcium phosphate (HG/HAp/TCP) scaffold loaded with VEGF can effectively promote cell adhesion, proliferation and migration [100]. In order to maintain the biological activity and continuous release of VEGF, Quinlan et al. incorporated alginate particles coated with VEGF into collagen HAp scaffold. The scaffold can continuously release VEGF for up to 35 days and produce more angiogenesis in the rat skull defect model [101]. In another study, calcium-deficient HAp particles (CDHAp) loaded with VEGF were prepared to improve the angiogenesis ability of scaffolds. Due to the hollow core of CDHAp, the initial burst of VEGF can be weakened and then slowly released, resulting in upregulation of angiogenesis-related genes [102].

#### 4.1.3. bFGF

Basic fibroblast growth factor (bFGF) is a growth factor and signaling protein encoded by the FGF-2 gene, which can promote division of mesoderm and neuroectodermal cells and has a strong angiogenesis effect. It promotes the growth of capillaries in new bone tissue [103]. In addition, bFGF has also been shown to promote healing of bone defects in osteoporotic animals [104]. Zhao et al. combined D-RADA16 self-assembled peptide hydrogels with n-HAp/polyamide 66 (PA66) scaffolds to control the release of bFGF. The bFGF can be continuously released for more than 168 h and accelerate the rate of bone healing [105]. Another study filled the bFGF-binding RADA16 peptide hydrogel into CaSO_4_/n-HAp bone cement, and the resulting composite biomaterial was able to significantly upregulate the expression of osteogenic markers [106]. Furthermore, bFGF can induce differentiation of periodontal ligament cells. Wang et al. combined the bFGF-loaded n-HAp-modified collagen material with Geistlich Bio-Gide (GBG) membrane to increase bFGF load rate, ultimately realizing regeneration of alveolar bone and cementum [107]. Kim et al. synthesized mineralized HAp/PCL nanofiber scaffolds loaded with bFGF to achieve continuous release of bFGF for 14 days, which effectively improved the proliferation and adhesion of rat mesenchymal stem cells [108]. In addition, another research team realized continuous release of bFGF from n-HAp/collagen (n-HAp/COL) scaffold for 17 days. Finally, the bFGF/n-HAp/COL scaffold promoted bone regeneration of rabbit mandibular defects [109].

#### 4.1.4. Combined Application of Growth Factors

Local delivery of multiple bone-inducing growth factors and angiogenic factors is a promising strategy to promote bone regeneration. Proper combinations and continuous release of growth factors can successfully induce stem cells and osteoblasts in bone defect areas [110]. Among them, BMP-2 is often used in combination with VEGF to achieve the effect of vascularized bone regeneration. Wang et al. first prepared PLGA microspheres loaded with VEGF and BMP-2 and then composited with n-HAp and collagen to form VEGF/BMP-2/nHAC/PLGAs three-dimensional scaffolds. The scaffolds loaded with both VEGF and BMP-2 had higher expression levels of osteogenic-differentiation-related factors than the scaffolds loaded with a single growth factor, which may be related to activation of the p38 MARK pathway causing Osterix phosphorylation [111]. Another study loaded SF microspheres with VEGF and BMP-2 on SF/n-HAp scaffolds. Rapid release of VEGF combined with the slow and sustained release of BMP-2 enables complete healing of rat calvarial defects 12 weeks after scaffold implantation [112]. BMP-2-derived peptide and QK (a VEGF mimetic peptide) were dually grafted to PA66 polymer chains to prepare peptide-decorated HAp/PA66/BMP-2/QK scaffolds to enhance bone formation after severe femoral fracture (periosteum scraped off) in SD rats [113]. Furthermore, BMP-2 can also be combined with bFGF for synergistic and additive effects. One study used n-HAp/collagen composite scaffolds for dual delivery of bFGF and BMP-2. Compared with pure bFGF or BMP-2, the bFGF/BMP-2/n-HAp/COL scaffold group had better cytocompatibility and osteogenic differentiation ability [114]. Another study used graphene oxide (GO) to immobilize bFGF and BMP-2 on PLGA/HAp nanofiber scaffolds and showed that the fabricated PLGA/HAp/GO/bFGF/BMP-2 composite scaffolds supported in vitro cell adhesion, proliferation and osteogenic differentiation of MC3T3-E1 [115]. Table 2 summarizes the growth-factor-loaded n-HAp composites for bone regeneration.

### 4.2. Polypeptides

In order to be better used as biomaterials for repairing bone defects, it is necessary to further improve the biological activity and osteogenesis of n-HAp-based composites. Combining peptides with biomaterials is a widely used method to improve the biological activity, biocompatibility and interaction with cells [116,117]. D-RADA16-RGD self-assembly peptide can be spontaneously assembled into nanofibers, which shows remarkable biological activity in promoting bone regeneration. Zhao et al. added D-RADA16-RGD to n-HAp/PA66 scaffold materials, which demonstrated that n-HAp/PA66/DRAA16-RGD composites had excellent bioactivity, which may be related to the hydrophobic alkyl tail of DRAA16-RGD interacting with cells or proteins [118]. γ-PGA is a natural anionic peptide with γ-linked glutamic acid, and its carboxyl and amide groups can be complexed with various metal ions, which has been widely used as tissue engineering materials because of its hydrophilicity, anti-inflammatory and immunomodulatory properties [119,120]. A research group synthesized γ-PGA modified HAp materials containing Cu^2+^, which realized controlled release of Cu ions [121]. Parathyroid hormone (PTH) peptide PTH (1–34) is the 1-34 amino acid fragment of parathyroid hormone, which targets cartilage and bone cells and regulates the balance of calcium and phosphorus. Previous studies have confirmed that intermittent use of PTH (1-34) can improve the fixation of implanted osteogenic materials in osteoporotic bone tissues and promote bone repair [122]. Zou et al. prepared Gel/n-HAp/PTH scaffold. With an increase in PTH (1-34) content, the cell adhesion on the scaffold surface increased, which was more conducive to bone regeneration [123]. Table 3 summarizes the polypeptide-loaded n-HAp composites for bone regeneration.

### 4.3. Vitamins

Vitamin D3 (VD3), also known ascholecalcin, is a regulator of extracellular calcium and phosphorus concentration. It plays a vital role in bone homeostasis by regulating the differentiation, proliferation and apoptosis of osteoblasts as well as the expression of specific bone proteins and growth factors in nascent bone tissue [124,125]. In one report, Sumathra et al. prepared a VD3-loaded cellulose functionalized n-HAp mesoporous silica composite (VD3/C/HAp/MSNS-3). At 20 days, VD3 was uniformly released with a release rate of 75.32%. Compared with pure C/HAp/MSNS-3 composite scaffold, this scaffold has better biocompatibility because it can promote the attachment, diffusion and proliferation of MG63 and improve the expression levels of genes, such as runt-related transcription factor 2 (Runx2), ALP and OCN [126]. According to another report, due to the hydrophobic property of VD3, Fayyazbakhsh et al. coated VD3 with gelatin and bonded it to layered double hydroxide–hydroxyapatite nanocomposites. Under the conditions of 37 ℃, PBS or pH = 5.6, the explosive release of VD3 was detected in the first 2 h, and then it continued to release steadily for 48 h, with a cumulative release of 16 days. The addition of VD3 resulted in an increase in Young’s modulus and negative Zeta potential of the scaffold and a 16% reduction in particle size, which might be due to the formation of hydrogen bonds between VD3 and other components [127]. Ignjatovi et al. synthesized nanocomposites containing VD3, hydroxyapatite and PLGA and implanted them into the mandibular defect area of osteoporosis rats. Six weeks later, obvious new bone, bone cement lines and blood vessels appeared in this area [128].

Vitamin K (VK) is mainly divided into two forms: phylloquinone (vitamin K_1_, VK_1_) and menaquinone (vitamin K_2_, VK_2_). Among them, VK_2_ can induce glutamic acid residue (Glu) of osteocalcin to form γ-carboxyglutamic acid residue (GLa), so it can delay bone loss and improve bone strength, which is considered to have the potential of bone tissue engineering application [129,130,131]. A research group has synthesized biomimetic hydroxyapatite (HAp)/xylitol adipate (PXSA) nanocomposite scaffold containing VK. The encapsulation efficiency of VK is about 80.3%, and the sustained release of VK is about 50.87% after 10 days. The HAp/PXSA/VK scaffold significantly promoted cell proliferation, adhesion and differentiation when MG63 was cultured on this scaffold [132]. Suzuki et al. prepared polylactic acid/hydroxyapatite core-shell microspheres loaded with VK_1_, in which polylactic acid is the core and hydroxyapatite is the shell. The material has a diameter of 40 to 80 nm, and the particle size increases with an increase in vitamin K_1_ loading. In vitro experiments confirmed its good drug loading capacity and pH sensitivity, and its drug loading value was about 250% [133]. One research group used vitamin E (VE) as a porogen and surfactant to optimize the porosity and permeability of collagen/n-HAp/β-TCP scaffolds. VE successfully increased the pore size, pore interconnection and surface permeability of scaffolds [134]. Table 4 summarizes the vitamin-loaded n-HAp composites for bone regeneration.

## 5. Drug-Loaded n-HAp Composite Scaffolds

**Figure 3:**
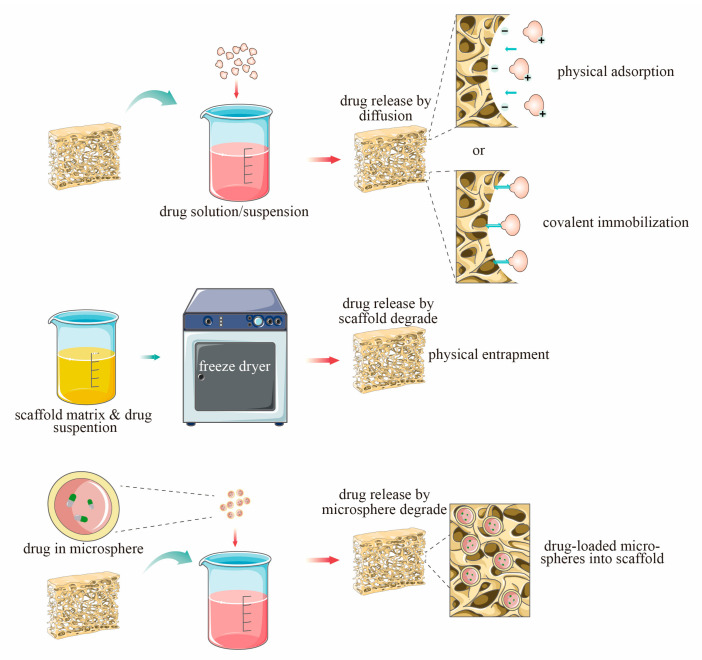
Loading methods of drugs on n-HAp scaffold.

Bone defects caused by some bone-related diseases, such as osteomyelitis, osteosarcoma, osteoporosis and bone tuberculosis, require not only surgery to repair the defect but also large doses of medication. Due to the unique structure of bone, delivering adequate doses of drugs into damaged bone tissue has been a challenge. In the traditional systemic route of administration, the drug is distributed throughout the body through the blood, which has the disadvantages of systemic toxicity, dysbacteriosis, poor targeting of target tissues and low patient compliance. Topical delivery systems can reduce side effects and increase drug concentrations in targeted tissues. Controlled release systems can also deliver drug molecules at a controlled rate and time to improve the efficiency of drug uptake by tissues. Some researchers have combined n-HAp composite bone scaffolds with drugs to make drug-loaded scaffolds that can release drugs locally and slowly and promote formation of new bone in the defect area, providing a new option for treatment of bone diseases. Drug-loaded bone scaffolds should provide suitable structures for bone regeneration and support and protect drugs from environmental degradation factors. Drug loading can be achieved by direct mixing with the scaffold material prior to manufacture by encapsulating the base drug carrier and adding the system to the scaffold material, by coating the prepared scaffold in a polymer or composite solution and by impregnating the prepared scaffold bracket to obtain (Figure 3).

According to the drugs contained in the n-HAp composite bone scaffold, the drugs can be mainly divided into antibacterial, antitumor, antiosteoporosis and antituberculosis drugs.

### 5.1. Antibacterial Drugs

Osteomyelitis is a chronic bone infectious disease caused by bacteria, of which *Staphylococcus aureus* is the most common pathogenic pathogen, causing osteolysis and formation of bone defects [135,136]. The traditional treatment of chronic osteomyelitis is excision of necrotic bone and infected soft tissue and widespread systemic use of antibiotics [137]. However, long-term systemic antibiotic therapy can lead to significant systemic toxicity and insufficient local drug concentrations. In recent years, use of biodegradable scaffolds loaded with antibacterial drugs is a common strategy to treat infectious bone defects with local antibiotics [138]. The local sustained-release antibiotic system can significantly improve the bioavailability of antibiotics without increasing the blood drug concentration, thereby reducing the systemic toxic and side effects of drugs [139]. The n-HAp composite scaffold has favorable biological activity and biocompatibility, can completely and effectively release the loaded antibiotics and provides a proper environment for new bone formation. Therefore, n-HAp is considered as one of the most promising biomaterials in drug delivery system for treating osteomyelitis [140,141]. Some recent studies on n-HAp composite scaffolds loaded with antibiotics are presented below.

Vancomycin is a glycopeptide antibiotic that interferes with cell wall synthesis by inhibiting peptidoglycan. It has minimal adverse effect on osteoblast and bone regeneration and has been widely used for treatment of bone tissue infection caused by methicillin-resistant *Staphylococcus aureus* (MRSA) [142]. After synthesis of the nano-composite fiber scaffold by Krishnan et al. [143], 5 wt% or 15 wt% vancomycin was loaded onto the scaffold by physical encapsulation (SE-V) and adsorption (SA-V) methods. In vitro tests demonstrated continuous release of vancomycin for 30 days. Furthermore, in vivo experiments indicated that the implanted scaffold could remove bacteria and promote new bone formation within 3 months. The ratio of large to small pores of the scaffold determines the drug release rate, with the large pores allowing rapid release and the small pores serving as a reservoir for sustained and long-term release. In one study, two osteoblastic filler scaffolds with different surface areas and porosities were prepared for vancomycin loading using polyurethane (PU), n-HAp, and acellular bovine bone particles. Scaffolds with high porosity have higher drug uptake and longer drug release time [144]. In another study, heparinized n-HAp/collagen biocomposites loaded with vancomycin were prepared using different sintering temperatures. Different sintering temperature may affect the release of vancomycin because of changing the granule size. At the 1050 °C, the material has high macroporosity and roughness. Vancomycin has been released continuously for 19 days in vitro and has strong antibacterial activity against attached bacteria and floating bacteria [140]. Lv et al. implanted vancomycin-loaded n-HAp/PLA composite into the chronic osteomyelitis (CO) mouse model. After 4 weeks, the vancomycin concentration was still higher than the minimum inhibitory concentration, which could effectively inhibit inflammatory reactions, such as congestion and edema, and effectively repair the bone defect [145].

Levofloxacin, a third class of fluoroquinolone antibiotics, exerts an antibacterial effect by inhibiting bacterial type II topoisomerase to prevent DNA replication and transcription [146]. A research group developed a new Levofloxacin/mesoporous silica microspheres/n-HAp/polyurethane (Lev/MSNs/n-HAp/PU) composite scaffold and conducted a series of studies. This scaffold has good biological safety and excellent antibacterial activity on both Gram-positive bacteria and Gram-negative bacteria. The results of drug release experiments showed that the drug in the scaffold could be released for up to 42 days [147]. Previous studies have found that Lev/MSNs/n-HAp/PU composite scaffold can effectively treat chronic osteomyelitis caused by *Staphylococcus aureus* in vivo and has good performance in repairing bone defects and controlling inflammation [137]. The results of experiments in vitro proved that this novel antibiotic-loaded biological composite scaffold had the dual functional potential of anti-infection and osteogenesis promotion. It can not only inhibit growth and adhesion of bacteria but also induce BMSCs to differentiate into osteoblasts, promote the proliferation of MC3T3-E1 and enhance the expression of OCN and type I collagen genes [148]. The results of these studies indicate that this composite antibacterial scaffold can be used as a drug delivery system for treating bone defects caused by chronic osteomyelitis.

The above research results indicate that combining antibiotics with n-HAp-based composite scaffolds not only inhibited bacterial adhesion and proliferation but also promoted new bone formation. The physicochemical properties of scaffolds (such as porosity), processing conditions and the concentration of antibiotics can affect drug release and osteogenic properties. With the growing problem of bacterial resistance, more research is needed to explore the optimal conditions for improving drug utilization efficiency.

### 5.2. Antitumor Drugs

Bone tumors are mainly divided into primary and secondary bone tumors. The most common of these primary bone tumors are osteosarcoma, chondrosarcoma and Ewing sarcoma, while secondary bone tumors can arise from various types of advanced cancer metastasis [149,150]. Traditional therapies for bone tumors have developed slowly, including surgery, chemotherapy, radiotherapy, hormone therapy and immunotherapy. These treatments have a series of disadvantages, such as multidrug resistance, tumor recurrence, severe side effects and large bone defects [151]. In order to overcome the shortcomings of the current treatment methods, bone-specific drug targeted therapy is an effective therapeutic strategy, which can concentrate the drugs on the tumor site, prolong the release time of the drugs and reduce the systemic adverse reactions. n-HAp is regarded as a good carrier for anti-tumor drugs due to its high affinity of surface and excellent osteogenic properties [152].

Doxorubicin (DOX), also known as adriamycin (ADM), is an anthracycline derived from streptomyces bacteria [153]. DOX is a usual antitumor drug that inhibits DNA replication and RNA transcription by interfering with the intercalated double helix structure of DNA and disrupting synthesis of nucleic acids [154]. Rong et al. prepared ADM-coated poly (lactic-co-glycolic acid)-co-nanoparticles (ADM-PLGA-NP) and loaded them onto porous n-HAp/collagen scaffolds. ADM-PLGA-nHAC composite scaffold has a pore diameter of 100–200 μm, a porosity of 82%, good pore connectivity and sustained and prolonged release of ADM within 28 days. Compared with direct intraperitoneal injection of ADM, this kind of drug-loaded scaffold implanted in nude mouse model can significantly inhibit growth of osteosarcoma cells, improve the anti-tumor effect and reduce adverse reactions [155]. In another study, to better control the drug release mode, Luo et al. intercalated doxorubicin into layered n-HAp and then mixed with PLGA to electrospin to fabricate DOX@LHAp/PLGA composite scaffolds. The results showed that DOX@LHAp/PLGA exhibited better drug carrier application potential compared with DOX@n-HAp/PLGA scaffolds made with needle-like n-HAp and greatly relieved the initial burst release of DOX, which may be related to intercalation of DOX into LHAp channels [156]. In order to make the developed DOX@LHAp/PLGA scaffold that has excellent osteogenic properties, the research group coated the surface of the DOX@LHAp/PLGA scaffold with a layer of polydopamine (PDA). PDA coating not only improves the hydrophilicity and mechanical properties of the material but also prolongs the drug release time and inhibits the growth of tumor cells and promotes the proliferation of MC3T3-E1 cells. In addition, in vivo experiments in mice show that coating PDA on DOX@LHAp/PLGA scaffold can greatly promote bone growth [157].

Metformin, an organic compound, is a first-line drug for treatment of type 2 diabetes. Metformin has been widely studied for its anticancer, anti-inflammatory and osteogenic effects [158,159]. Tan et al. successfully developed the poly (L-lactic ac-id)/n-HAp/metformin (PLLA/n-HAp/MET) nanocomposite scaffold using SLS technology. The scaffold induces cell apoptosis through the mitochondrial apoptotic pathway, inhibits tumor cell proliferation and blocks the cell cycle in G2/M phase [1]. Zoledronic acid is a nitrogen-containing bisphosphonate that is widely used in clinical practice for its ability to relieve the pain of patients with primary osteosarcoma and bone metastases. Lu et al. [160]. developed a novel zoledronic-acid-loaded CS/n-HAp composite scaffold with an in situ precipitation method, which showed good anti-tumor performance for giant cell tumor of bone (GCT). The prepared CS/n-HAp/Zol scaffold also has excellent antibacterial activity, which is mainly attributed to chitosan [161].

Both primary bone tumors and metastatic bone tumors require prompt treatment and a combination of therapeutic approaches. The above research results show that the combination of n-HAp composite scaffolds and chemotherapeutic drugs can not only effectively inhibit bone tumors but also repair the bone defects caused by tumor resection. It is believed that this treatment strategy will eventually bring benefits to patients with bone tumors.

### 5.3. Anti-Osteoporotic Drugs

Osteoporosis is a bone metabolic disease caused by many factors, which can be divided into primary postmenopausal osteoporosis, senile osteoporosis and secondary osteoporosis. Its clinical profile includes decreased bone mineral density and bone mass, destruction of bone microarchitecture and increased bone fragility [162]. Osteoporosis can lead to excessive decalcification of osteoclasts and decreased phenotypic expression of osteoblasts, often showing impaired healing [163]. Current treatments for osteoporosis include taking bisphosphonates, calcitonin and other drugs [164]. However, this systemic treatment method suffers from low drug bioavailability and large side effects. Currently, local controlled release drugs in bone defect areas have been shown to be beneficial for the treatment of osteoporotic bone defects.

Bisphosphonates, a carbon-substituted analog of pyrophosphates, are the drug of choice for prevention and treatment of osteoporosis. Bisphosphonates have high affinity for hydroxyapatite in bone, which can not only inhibit the activity of osteoclasts but also promote the proliferation and maturation of osteoblasts [165]. Rajan et al. used electrostatic spinning technology to load pamidronate into polycaprolac-tone/polycaprolactone-polyethyleneglycol-polycaprolactone/n-HAp (PCH) scaffold, and the study confirmed that PDS in the composite scaffolds was well-released in phosphate buffer. The PCH scaffolds incorporating 3 wt % PDS can effectively promote the bone healing of calvarial defects in osteoporotic rats [166]. Alendronate is a nitrogen-containing bisphosphonate. It is widely used for treatment and prevention of osteoporosis due to its ability to inhibit osteoclast activity, increase the anabolic activity of osteoblasts, stimulate BMP-2 and enhance osteogenesis of human-adipose-derived stem cells [167]. Wu et al. embedded CS/n-HAp microspheres loaded with alendronate in PLLA/n-HAp scaffolds and developed a microsphere-scaffold hybrid system. The scaffolds increased the ALP activity and calcium deposition of adipose-derived stem cells (ASCs) and completely repaired the large radial defect in rabbits within 8 weeks [28].

In addition to bisphosphonates, several other drugs have been studied for treatment of osteoporotic bone defects by local delivery. Calcitriol, the active form of vitamin D3, is a well-recognized lipid-soluble small molecule against osteoporosis [168]. Hu et al. studied a hybrid hydrogel system containing calcitriol. Owing to the triple sustained-release effect of micelles, dopamine-modified n-HAp and hydrogel calcitriol can be released continuously for more than 35 days. In vitro cell culture experiments, the proliferation, migration and osteogenic differentiation of bone mesenchymal stromal cells of ovariectomized rats (OVX-rBMSCs) were successfully promoted [169]. Resveratrol is an anti-inflammatory small molecule with multiple functions, such as anti-oxidation, anti-inflammatory and anti-tumor. It can promote osteoblast differentiation and inhibit osteoclastogenesis by inhibiting tumor necrosis factor-α [170,171]. Li et al. prepared n-HAp/resveratrol/CS (n-HAp/Res/CS) composite microspheres by emulsion chemical cross-linking method, which could create an anti-inflammatory microenvironment and promote osteogenesis through local sustained release of Res. In vivo and in vitro experimental results show that the microspheres can reduce the expression of tumor necrosis factor-α (TNF-α), interleukin-1 (IL-1) and inducible nitric oxide synthase (iNOS) in RAW264.7 cells, promote the proliferation and osteogenic differentiation of bone marrow mesenchymal stem cells and promote cartilage growth and bone remodeling in the femoral condyle defect area of osteoporoticrats in vivo [172]. Naringin, a flavonoid commonly found in citrus peels, has improved bone mass and anti-osteoporosis effects [173]. Yu et al. prepared naringin/gelatin microspheres/n-HAp/SF (NG/GMS/n-HAp/SF) composite scaffolds by freeze-drying. Naringin can be continuously released in the scaffold for more than 6 days. The composite scaffold could promote the proliferation, adhesion and expression of bone-specific genes of BMSCs, and at it could completely repair the bone defect 16 weeks after implantation into the vertebral defect area of osteoporotic rats [174].

The main mechanism of action of anti-osteoporosis drugs is to restore the dynamic balance of bone resorption and bone formation by reducing the activity of osteoclasts or increasing the activity of osteoblasts. n-HAp composites have certain osteogenic properties. Encapsulation of anti-osteoporosis drugs in n-HAp complexes can target the drugs to the sites of osteoporotic bone defects, achieve slow-release control, reduce the wastage of drugs and the adverse reactions of patients.

### 5.4. Anti-Tuberculosis Drugs

Bone tuberculosis is a devastating disease caused by *mycobacterium tuberculosis* invading bones or joints. It most often occurs in the spine and other weight-bearing bones. Lesions will form cold abscesses and caseous necrosis, leading to bone destruction [175,176]. Treatment is usually surgical debridement combined with anti-tuberculosis drugs. However, due to poor blood circulation at the lesion site, it is difficult for anti-tuberculosis drugs to reach the lesion site through the blood, resulting in too low local drug concentration and incomplete killing of residual *mycobacterium tuberculosis* [177,178]. Loading anti-tuberculosis drugs into n-HAp composites by various drug loading techniques can effectively improve the efficiency of anti-tuberculosis treatment and provide an effective method for the treatment of tuberculous bone defects.

Anti-tuberculosis drugs can be divided into two categories: first-line anti-tuberculosis drugs and second-line anti-tuberculosis drugs. Among them, first-line anti-tuberculosis drugs are the most commonly used drugs in clinical practice, mainly including rifampicin, isoniazid, pyrazinamide and ethambutol [179]. Among them, rifampicin is a hydrophobic anti-tuberculosis drug belonging to the rifamycin family, which has a strong bactericidal effect on *Mycobacterium tuberculosis* by inhibiting formation of bacterial RNA [180]. Shi et al. used HAp/CS bone cement to load rifampicin (RFP). The addition of RFP increases the porosity of bone cement and decreases its compressive strength, but its compressive strength is higher than that of cancellous bone. The bone cement released 84.7% of RFP within 31 days, and the concentration of RFP remained above the minimum inhibitory concentration after 31 days, allowing for antituberculosis drug delivery [181]. The mechanism of action of isoniazid against tuberculosis is to inhibit synthesis of mycolic acid in *mycobacterium tuberculosis*, resulting in cell wall rupture. Due to its good biofilm permeability and good efficacy, it is classified as the anti-tuberculosis drug of choice. Xie et al. loaded isoniazid onto n-HAp/PA66 scaffolds using chitosan and glutaraldehyde. The porosity of the composite scaffold is 58.9%, and isoniazid in the scaffold can be effectively released in the PBS dialysis bag for 15 days and in rabbit femoral condyle for 28 days. The scaffold can inhibit the proliferation and biofilm formation of *mycobacterium tuberculosis* and can promote new bone formation in vivo [182]. Since the clinical combination of RFP and isoniazid has a synergistic effect and can delay the drug resistance of bacteria, Qayoom et al. used a sulphate HAp carrier to locally deliver RFP and isoniazid and evaluated the interaction between n-HAp and anti-tuberculosis drugs. The results show that the surface binding energy between RFP and n-HAp is higher than that of isoniazid, resulting in a longer release time of RFP. The combined use of RFP and isoniazid can better inhibit the biofilm formation of pathogenic bacteria and can promote migration of osteoblasts to the surface of the material, facilitating repair of bone defects [183]. In another research group, κ-carrageen grafted/n-HAp (κ-Car-MA-INH/n-HAp/RFP) composites loaded with RFP and isoniazid were prepared. The composites are spherical with a diameter of 250 nm, which is a good drug carrier and can easily flow in biological liquid. The entrapment efficiency of RFP and isoniazid is 84% and 72%, respectively, which may be due to the fact that isoniazid is hydrophilic while RFP is hydrophobic. The composites showed significant cell proliferation promotion, higher antibacterial activity, no toxicity and protected bones from the influence of bacterial environment [184].

In clinical practice, the combination of four common anti-tuberculosis drugs is often used to treat tuberculosis, also known as quadruple therapy. However, in most of the current studies, only one or two anti-tuberculosis drugs are loaded on the drug-loaded scaffold. Continued use of a single drug may increase drug resistance of *mycobacterium tuberculosis*, leading to relapse of tuberculosis. Therefore, loading more than two anti-tuberculosis drugs on scaffolds should be studied more extensively in the future. Table 5 summarizes the drug-loaded n-HAp composites to promote bone regeneration.

## 6. Loading Mode of n-HAp Composite Scaffolds

n-HAp composites are often combined with various osteoinductive biological factors or drugs to improve their osteoinductive and angiogenesis activities. However, different loading modes will affect the loading rate and release efficiency of bioactive factors and drugs. In this section, it mainly summarizes three common loading methods, namely physical bonding, coating and microsphere loading.

### 6.1. Physical Combination

Common methods of loading biological factors include physical adsorption, soaking, drying and coprecipitation. The pure physical adsorption operation is simple, and the biological activity of the scaffold can be protected without secondary sterilization [182,185]. Du et al. covered the n-HAp/coral scaffold with rhVEGF_165_ by direct physisorption. Experimental results showed that this scaffold could only improve early angiogenesis without significantly increasing bone regeneration, indicating that the release rate of rhVEGF_165_ was unsatisfactory, and, thus, it could not be sustained and fully functional [186]. Chen et al. prepared HACS composite porous scaffold with HAp, CS and PCL. Then, they soaked the scaffold in 12.5 µg/mL VEGF solution and finally freeze-dried to achieve the surface modification of VEGF. The loading of VEGF increased with an increase in soaking time. VEGF released by HACS scaffold was 1.42 µg/mL and 3.04 µg/mL respectively, after soaking for 1 day and 14 days [98]. In addition, Hu et al. combined BMP-2 and bFGF on n-HAp/collagen composite scaffold by mechanical mixing and freeze-drying to form a bFGF/BMP-2/n-HAp/Col scaffold. Sudden release of growth factors in the scaffold occurred from 3 to 5 days and stabilized after 5 days. bFGF and BMP-2 could be released for 17 days and 19 days, respectively [114]. However, the disadvantage of physical adsorption is that the connection between drug and scaffold is weak, with burst release, making it difficult to meet the demand of prolonged drug release. To solve these problems, Liu et al. immobilized VEGF on PCL/HAp scaffolds by coprecipitation with apatite. This biomimetic coprecipitation method has mild reaction conditions, and VEGF is incorporated into the mineralized apatite layer during coprecipitation with moderate bonding strength [99].

### 6.2. Coating

Recently, mussel-inspired polydopamine (PDA) coating has attracted wide attention for its unique adhesion [187]. PDA can be tightly bound to different surfaces by covalent and noncovalent interactions, so it can be used as an intermediate to immobilize biomolecules onto various natural or synthetic scaffold surfaces [188,189]. Zhou et al. successfully bonded BMP-2-derived peptide (P24 peptide) to n-HAp/RHLC/PLA scaffold by PDA-assisted coating. The efficiency of PDA-assisted immobilization of P24 peptide is 90 ± 6.53%, while the physical adsorption immobilization efficiency of P24 peptide is only 48 ± 3.03%. The results showed that the PDA coating had higher immobilization efficiency compared to physical adsorption [190]. Zhao et al. also effectively fixed BMP-2 on PDA-PLGA/HAp fiber scaffold through PDA coating and realized the controlled release of BMP-2. Meanwhile, PDA modification improved the hydrophilicity of the scaffold [191]. In addition, Ye et al. used a PDA-assisted coating strategy to immobilize BMP-2-derived peptides on 3D scaffolds to obtain n-HAp/PLA/gelatin-peptide (n-HAp/PLA/GEL-PEP) nanofiber scaffolds. The immobilization efficiency of BMP-2 polypeptide by PDA reached 89.62 ± 3.07%, and the release rate of BMP-2 polypeptide decreased continuously in the following 21 days. However, the immobilization efficiency of physical adsorption was only 49.71 ± 3.43%, the burst release rate on the first day was 36.4% and the accumulated polypeptide release within 7 days was 87.2% [92]. The above experimental results show that the mussel-inspired PDA coating-assisted strategy improves the immobilization efficiency of biological factors and effectively reduces its release rate. Heparin-bound biomaterials have been demonstrated to increase growth factors loading by reversibly electrostatically binding growth factors. Yan et al. heparinized the surface of Sr-n-HAp/SF scaffold and loaded BMP-2. The experimental results showed that the scaffold could effectively maintain the release of BMP-2 and promote proliferation and osteogenic differentiation of bone marrow mesenchymal stem cells [90]. The electrostatic interaction of electronic groups on the surface of graphene (GO) and the hydrophobic region of its core can enhance its binding force with growth factors. Therefore, GO can be used as an intermediate to couple growth factors to the surface of composite scaffolds. Ren et al. fixed bFGF and BMP-2 on PLGA/HAp scaffold through GO coating, and the binding efficiency of bFGF and BMP-2 to PLGA/HAp/GO nanofiber scaffold is more than twice that of PLGA/HAp nanofiber scaffold [115]. Due to the low hydrophilicity and lack of functional groups on the surface of many osteogenic scaffolds, the loading rate of biological factors is low. The auxiliary fixation of the coating can provide multifunctional and multipurpose surface modification and improve the loading rate of biological factors. Therefore, application of various coatings deserves attention.

### 6.3. Microsphere Loading System

In recent years, microspheres have shown great potential as a drug delivery system in bone tissue engineering. This method can reduce the loss of bioactive substances, especially water-soluble substances, improve their effective utilization rate at the local administration site and achieve controlled release of drugs. At the same time, combining the microspheres with the scaffold can maintain good local treatment and sufficient mechanical strength [192]. Li and his colleagues functionalized CS/n-HAp scaffold with simvastatin (SIM)-loaded PLGA microspheres by combining a freeze-drying technique with a modified water–oil–water emulsion method, and the encapsulation efficiency of SIM was 85.6%. The results of UV spectrophotometry showed that 37.16% of SIM was released in the first 3 days, and then SIM was slowly released for 30 days [193]. Shen et al. loaded BMP-2 into SF microspheres and then covered the microspheres and stromal-cell-derived factor-1 (SDF-1) on SF/n-HAp scaffolds through physical adsorption to achieve controlled release of two bioactive molecules in sequence. The initial burst release of SDF-1 promoted recruitment of MSCs, and then BMP-2 slowly and continuously released for up to 3 weeks, inducing osteogenic differentiation of MSCs [194]. Furthermore, one research group prepared loaded salvianolic acid B (Sal B) coated with CS microspheres by emulsification method and then immobilized it on the surface of HAp scaffold pre-coated with alginate. The optimum alginate coating concentration of 1% can increase the compressive strength of the scaffold without affecting the porosity and increase the adhesion rate of the microspheres due to the electrostatic effect. The CS microspheres containing Sal B had an early burst on day 2 and the remaining Sal B was released slowly and continuously for the remaining 30 days to 60% of the total dose [195]. In addition, another research group prepared CS microspheres encapsulating polypeptide P24 (CMs-P24) by emulsion method and then prepared n-HAp/collagen/poly (L-lactic acid)/CS microspheres (NaAC/PLLA/CMs) composite scaffold by thermally induced phase separation method. This scaffold has the ability to control the release of BMP-2 synthetic peptides. With an increase in CMs content, the degradation rate of the scaffold increased [196]. Microspheres are rapidly developing as a drug delivery system, which can provide long-term sustained and slow drug release effect and protect the activity of biological agents. However, the bonding methods between microspheres and scaffolds have an important impact on the loading efficiency and release effect of microspheres, which deserves further exploration.

Composite scaffolds functionalized with various bioactive factors have been investigated for many years. Researchers have used various methods to combine bioactive factors and drugs with scaffolds to achieve controlled release, which is conducive to bone regeneration. Among them, the physical adsorption is simple, but it is difficult to meet the demand for long-term drug release due to poor connection leading to burst release. In addition, auxiliary fixation effect of various coatings has been explored and applied. The functionalization of the coating on the scaffold surface can bring its own biological activity to the scaffold, improve the fixation of growth factors and achieve slow release. Currently, microspheres are widely used as carriers of drug delivery systems. Microspheres can encapsulate various types of drugs and prolong the drug release time, but there may be the problem of early delayed release. Therefore, various effective methods for biomaterials to control the release of bioactive factors deserve further exploration.

## 7. Conclusions and Prospects

This paper summarizes the latest progress of n-HAp composites loaded with bioactive factors and used as drug delivery systems for bone defect repair in recent years. Indeed, n-HAp has become an excellent material for bone tissue engineering due to its good biocompatibility, non-immunogenicity and non-toxicity. A combination of n-HAp and other materials can form a three-dimensional bone graft scaffold with sufficient mechanical strength, suitable pore size and porosity and osteoconductivity, which compensate for the shortage in mechanical properties of n-HAp. Due to n-HAp having good drug loading properties, the combination of bioactive factors and n-HAp composite scaffolds can overcome the shortcomings of insufficient osteoinductivity of some scaffold materials. Meanwhile, controlled release of bioactive factors is realized, and their activity is maintained. Moreover, the bone defects caused by bone diseases such as osteomyelitis, bone tumors, osteoporosis and bone tuberculosis require bone reconstruction and corresponding drug treatment. Therefore, the combination of n-HAp composite scaffolds with a number of drugs can not only play a role in supporting bone defects but also inhibit growth of bacteria, tumor cells and osteoclasts through slow-release drugs to achieve good bone healing effects. This sustained-release system makes n-HAp-based scaffolds a promising strategy for bone tissue engineering application.

Breakthroughs have been made in the study of n-HAp composites as bone scaffolds. However, there are still some concerns. For example, there is a certain gap between the mechanical strength or elastic modulus of the composites and the bone, the degradation rate cannot be consistent with the rate of new bone formation and the loading dose and the sustained release time of bioactive agents and drugs cannot meet requirements. With continuous development of composite artificial bone materials research, future research on repair of bone defects with n-HAp composites may focus on the following aspects: (1) using new preparation processes to improve n-HAp composite scaffolds’ mechanical strength and degradation rate. For example, with the help of 3D printing technology, composite scaffolds with different pore sizes and porosity can be designed to modify their physicochemical properties. (2) By modifying the surface of the composite scaffold or making drug-loaded nano- or micro-spheres to be adsorbed to the scaffold, the effects of increasing the loading rate of bioactive factors and slow-releasing drugs can be achieved. (3) Two or more bioactive factors and drugs are loaded on the n-HAp composite scaffold to produce a better therapeutic effect, for example, the combination of growth factors and antibiotics to regenerate new bone at sites of bone defects caused by infection.

In summary, n-HAp composite scaffolds can be a promising strategy to treat bone defects through modification by different kinds of bioactive factors or loading various drugs, thus accelerating healing of bone defects.

## Figures and Tables

**Table 1:** Summary of n-HAp composite scaffolds for bone regeneration.

Material	Scaffold	Feature	Reference
Chitosan	CS/n-HAp composite scaffold	Scaffolds with an n-HAp/CS concentration of 75/25 *w*/*w* have better physicomechanical characteristics An increased HAp concentration exceeding 80% has been reported to result in friable scaffolds in vitro	[52,54,55]
Silk fibroin	SF/n-HAp composite scaffold	The compressive strength increases with an increase in n-HAp concentration	[58] [60]
Polylactic acid	PLA/n-HAp composite scaffold	n-HAp can buffer the acidity of PLA degradation With an increase in PLA concentration, the porosity of the scaffold decreases	[70] [69] [63]
Polycaprolactone	PCL/n-HAp composite scaffold	The degradation rate of scaffold increases with an increase in n-HAp content It has antibacterial activity	[74] [77] [81]

**Table 2:** Summary of the growth factor-loaded n-HAp composites for bone regeneration.

Growth Factor	Scaffold	Release Profile	Outcome	Reference
BMP-2	n-HAp/PLA-PEG	BMP-2 sustained release for 21 days	Promotes bone regeneration in spinal fusion models	[17]
BMP-2	PHB-PDA	BMP-2 can be released continuously for 30 days	Cell attachment, proliferation and differentiation were significantly upregulated in vitro In vivo promotion of bone healing in rabbit calvarial defects	[88]
BMP-2	n-HG-SFD hydrogel	BMP-2 sustained release for more than 24 days	Promoted in vitro osteogenic differentiation and compatibility of rBMSCs Skull defect in mice repaired	[89]
BMP-2	Sr-n-HAp-SF	The BMP-2 from 10% Sr-n-HAp/SF-BMP-2 scaffold released approximately 80% by the 14th day	Osteogenesis-related genes upregulated in BMSCs Increased bone density in skull defects, new bone formation	[90]
BMP-2-derived peptides	n-HAp/PLA/Gel nanofibers	The release of the polypeptide continued until 21d	Promotes osteogenic differentiation of BMSCs in vitro and bone regeneration in a rat calvarial defect model	[92]
BMP2-mimicking peptide P28	nHAC/PLA	The release rate of P28 in 14 days is about 72 ± 3%	Promoted MC3T3-E1 proliferation, recruitment and differentiation in vitro Rabbit femoral condyle defect was repaired in vivo	[94]
BMP-6	n-HAp/Gel/GMS	BMP-6 was released for 20 consecutive days, and the cumulative release amount was about 95%	The composites can induce osteogenic differentiation of BMMSCs in vitro and promote formation of new bone in vivo	[84]
BMP-9	n-HAp/ColI/MWCNT	–	The 1%MWCNT group was the most suitable for bone tissue engineering BMP-9-loaded nHACM scaffolds exhibit good biocompatibility and osteogenic capacity	[97]
VEGF	HACS	The amount of VEGF released by the HACS scaffolds on days 1 and 14 was 1.42 μg/mL and 3.04 μg/mL	Promote proliferation of hBMSCs and HUVECs; improved regeneration and remineralization of bone tissue in rabbit femoral defect	[98]
VEGF	PCL/HAp	VEGF was released rapidly in the first 3 days and slowed down after 3 days	Enhanced osteogenesis of stem cells and preeminent vascularized bone regeneration	[99]
VEGF	HG/HAp/TCP	–	Increased expression of genes related to osteogenesis and angiogenesis and regeneration of new bone	[100]
BMP-2 and VEGF	SF/n-HAp	BMP-2 released 6.7% in the first day and 15.5% in 28 days VEGF released 48.7% in the first day and 66.4% in 10 days	Promoted adhesion and proliferation of MC3T3-E1 cells and accelerated formation of vascularized bone in rats with calvarial defects	[112]
BMP-2-derived peptide and QK	n-HAp/PA66	n-HAp/PA66-B1+Q1 released about 81.07 ± 3.27% of QK and 78.56 ± 3.66% of BMP-2-derived peptides within 15 days	Promoted proliferation and differentiation of rBMSCs and HUVECs and bone formation after severe femoral fracture (periosteal scraping) in SD2 rats	[113]
bFGF	n-HAp/PA66	The n-HAp/PA66/D-RADA16/bFGF scaffold released approximately 50% of bFGF within 48 h with no apparent burst release	Promoted proliferation, adhesion and osteogenesis of bone marrow mesenchymal stem cells and bone repair of rat femoral defects	[105]
bFGF	CaSO_4_/n-HAp	bFGF is released suddenly within 8 days, with a slow and sustainable release over 32 days	Improved in vitro osteogenic differentiation of MC3T3 cells and in vivo bone formation in femoral bone defects	[106]
bFGF	nHAC/bFGF-GBG	–	Promoted HPDLC cell growth and attachment and growth of new alveolar bone Increased ALP expression and calcium nodule formation by the BMSCs	[107]
bFGF and BMP-2	n-HAp/COL	The cumulative release days of bFGF and BMP-2 were 17d and 19d, respectively, and the amount of factor released was 91.05 ± 3.38% and 90.05 ± 2.08%, respectively	Promotes BMSCs adhesion, proliferation and ALP expression	[114]
bFGF and BMP-2	PLGA/HAp	–	Promoted adhesion, proliferation and osteogenic differentiation of MC3T3-E1 cells, and increased ALP activity and osteogenesis-related gene expression levels	[115]

**Table 3:** Summary of polypeptide-loaded n-HAp composites for bone regeneration.

Polypeptide	Scaffold	Feature	Function	Reference
RADA16-RGD	n-HAp/PA66	Self-assembled nanofibers by intermolecular hydrogen bonding	Promote cell proliferation, differentiation, adhesion and migration	[118]
γ-PGA	γ-PGA/Cu_x_HAp	Forming network complex to control the release of metal ions	Control the release of copper ions and have angiogenesis, osteogenesis and antibacterial activities	[121]
PTH (1–34)	PTH (1–34)/n-HAp /CS/SA	With an increase in PTH (1–34), the nanofibers of the scaffold become thinner and more conducive to osteogenesis	Promote osteogenic differentiation of rBMSCs and enhance cell adhesion	[123]

**Table 4:** Summary of vitamin-loaded n-HAp composites for bone regeneration.

Vitamin	Scaffold	Load Method	In Vitro Drug Release	Reference
VD3	C/HAp/MSNS-3	Freeze drying	The release amount reached 75.32% within 20 days	[126]
	LDH-HAp/gelatin	Gelatin coating	Bursting release within the first 2 h, stable release within 48 h, slow release until 16 days	[127]
	HAp/PLGA	Microsphere loading	–	[128]
VK	HAp/PXSA	Freeze drying	The release amount reaches 50.87% within 10 days	[132]
VK_1_	PLA/HAp	Microsphere loading	The release amount increases with the decrease in pH value of phosphate buffer solution	[133]

**Table 5:** Summary of n-HAp composites as drug carriers to promote bone regeneration.

Drug	Scaffold	Cell/Animal	Function	Reference
Vancomycin	Silica-coated n-HAp/gelatin reinforced with PLLA yarns	MSCs/Wistar rats	Remove bacteria, promote new bone formation	[143]
	HAp/PU/ decellularized bovine bone particles	Rabbit model of post-surgical OM	Promotes bone formation, prevents infection in bone defects	[144]
Levofloxacin	MSNs/n-HAp/PU	Rabbit model of chronic osteomyelitis	Repair bone defect, inflammation controlled	[137]
		L929 cells	Perfect antibacterial activity, no cytotoxicity, biosafety	[147]
		BMSCs/MC3T3-E1	Promote osteogenic differentiation of MSCs, proliferation of MC3T3-E1 cells and inhibit apoptosis	[148]
Doxorubicin	n-HAp/collagen	MG63 cells/New Zealand rabbits/ Sprague–Dawley rats/tumor-bearing nude mice model	Sustained release drug properties, inhibited tumor cell growth, bone repair	[155]
	LHAp/PLGA	MG-63 cells/ MC3T3-E1 cells/mice	Inhibits tumor cell growth, improves osteoblast adhesion and proliferation and promotes new bone formation	[157]
Metformin	PLLA/n-HAp	BMSCs/Saos-2 cells	Induces apoptosis of osteosarcoma cells, promotes osteogenic differentiation of cells	[1]
Zoledronic acid	CS/n-HAp	GCTBs/hBMSCs	Upregulates pro-apoptotic genes, downregulates osteoclast genes, induces osteogenesis, inhibits bacterial growth	[136]
Pamidronate	PCH	Osteoporosis rat animal model	Accelerates healing of osteoporotic bone defects	[166]
Alendronate	PLLA/n-HAp	ASCs/Rabbit radius defect model	Improves ALP activity and calcium deposition, repairs large bone defects	[28]
Calcitriol	Gel/HA-D/M	BMSCs/Rat critical-size femoral defect model	Promotes cell proliferation, migration and differentiation, healing of femoral defects	[169]
Resveratrol	n-HAp/CS	RAW264.7 cells/BMSCs/SD rats	Anti-inflammatory, promotes cell osteogenic differentiation, bone regeneration and endochondral growth	[172]
Naringin	GMs/n-HAp/SF	BMSCs/SD rats	Promotes BMSCs adhesion, proliferation and calcium nodule formation, new bone formation	[174]
Rifampicin	CS/n-HAp	MC3T3-E1 cells	Long-term controlled drug release, anti-tuberculosis, no cytotoxicity	[181]
Isoniazid	n-HAp/PA66	BMSCs/New Zealand rabbits	Inhibition of *mycobacterium tuberculosis* activity, good osteoconduction and osseointegration properties	[182]

## Data Availability

Not applicable.
